# Antitumor activities of Liver-targeting peptide modified Recombinant human Endostatin in BALB/c-nu mice with Hepatocellular carcinoma

**DOI:** 10.1038/s41598-017-14320-0

**Published:** 2017-10-26

**Authors:** Ma Yan, Bao Dongmei, Zhang Jingjing, Jin Xiaobao, Wang Jie, Wang Yan, Zhu Jiayong

**Affiliations:** 1 Guangdong Provincial Key Laboratory of Pharmaceutical Bioactive Substances, School of Basic Courses, Guangdong Pharmaceutical University, Guangzhou, 510006 People’s Republic of China; 2 Pharmacy, Shexian People’s Hospital, Huangshan, Anhui 245200 People’s Republic of China

## Abstract

In our previous study, a liver-targeting peptide CSP I-plus modified recombinant human Endostatin (rEndostatin, endostar) (rES-CSP) was constructed and showed potent antiangiogenic capability and could specifically bind to human hepatocellular carcinoma cells to make a direct inhibition *in vitro*. In this study, the biological activities of rES-CSP *in vivo* were evaluated by subcutaneous and orthotopic xenograft nude mice model of human hepatocellular carcinoma cells HepG2. We found that rES-CSP significantly decreased tumor volume to 54.9% in the nude mice with subcutaneous xenograft compared with the control. In orthotopic xenograft model, rES-CSP not only decreased tumor volume (to 39.6% compared with the control) and tumor weight, it also increased its biodistribution in the liver tissue and hepatoma tissue. Moreover, lower microvessel density (MVD) and higher apoptotic index (AI) were also observed in the tumor tissues. It had no significant side-effects on the heart, liver, spleen, lung and kidney of mice. Results indicated CSP I-plus modified Endostar may be a potential candidate for a targeting therapy on hepatocellular carcinoma.

## Introduction

Hepatocellular carcinoma (HCC) is the fifth most frequently diagnosed cancer worldwide, and it is the second and sixth leading cause of cancer death in men and women, respectively^1,2^. Surgical resection and liver transplantation are the most effective method of treating HCC. HCC has a long latent period, coupled with the high recurrence rate after surgery, in which chemotherapy is often the only option^3,4^. However, chemotherapy is often with a low response rate and some severe side-effects. Therefore, the 5-year overall survival rate is poor^5^. HCC has an abundant blood vessels, which has been identified to play a critical role in tumor growth, invasion, and metastasis^6,7^. Inhibition of angiogenesis may be an effective strategy to improve the overall survivals.

Endostatin (ES) has been proved to inhibit the proliferation, migration, and tube formation of endothelial cells, thus eventually interrupting angiogenesis and tumor growth^8,9^. Aside from its antiangiogenic activity, ES exerts a direct anticancer action in certain tumor cells. It could bind to cells with alpha5 integrin, inhibit cells migration, invasion and induced apoptosis, and reduced the development of highly vascularized mammary tumors through down-regulation of VEGF expression^10–12^. The latest researches suggest ES also inhibits cell growth and migration by skewing macrophage polarity from a pro-tumorigenesis (M2) phenotype to a pro-immune (M1) phenotype^13,14^. Recombinant human Endostatin (rEndostatin, endostar), by the linkage of MGGSHHHHH to the N terminus stabilizes ES and has achieved favourable results combined with radiotherapy or chemotherapy in experimental and clinical research^15–17^.

It was reported that the therapeutic agents accumulating in the tumor could be as little as 5–10% of the dose accumulated in normal organs^18^. Limited accumulation of active therapeutic agents in the HCC site resulted in drug concentration too low to be efficacious for HCC. As such, there is a desperate need for a better treatment approach or drug delivery system to improve the therapeutic effect. Based on these, site-specific and targeted drug delivery system for HCC treatment are proposed, in which the HCC-specific ligands or antibody modified carriers are utilized. Although monoclonal antibodies have shown clinical potential as tumor targeting agents, they are limited by their large molecular size and poor tumor penetration^19^. These limitations can be overcome by using peptide ligands, which are smaller, less immunogenic molecules, and easier to produce and manipulate^20^.

Circumsporozoite protein (CSP) is the major coat protein on the *Plasmodium* sporozoite that displays highly specific and highly efficient targeting to liver, and is required for sporozoite development and invasion of hepatocytes^21,22^. CSP I-plus is N end of the CSP and could specifically bind to the liver which overexpress the highly sulfated heparan sulfate proteoglycans (HSPGs)^23,24^. In our previous study, the fusion protein was constructed with introducing the CSP I-plus sequence into the C-terminus of Endostar through bioengineering methods, and expressed in *Escherichia coli*. The biological activity tests showed that CSP I-plus modified Endostar (rES-CSP) inhibited the proliferation and migration of human umbilical vein endothelial cells (HUVECs) and showed potent antiangiogenic capability on HUVECs tube formation assay and chick embryo chorioallantoic membrane(CAM) assay^25^. Meanwhile, rES-CSP could specifically bind to the hepatocellular carcinoma cells HepG2 and made a direct inhibition on tumor cells *in vitro* ^26^. But the biological activities of rES-CSP on homing to hepatocellular carcinoma tissue and anti-HCC *in vivo* have unknown.

In order to study the effect of rES-CSP, subcutaneous xenograft and orthotopic xenograft of human hepatocellular carcinoma cells HepG2 were established in remale BALB/c nu/nu nude mice. The antitumor effect of rES-CSP was measured. The biodistribution of rES-CSP was analyzed by ELISA and immunofluorescence. Meanwhile, the microvessel density (MVD) and apoptotic index (AI) of the tumor tissues were detected by anti-CD31 immunohistochemistry and the transferase mediated deoxyuridine triphosphate (dUTP)-digoxigenin nick end-labeling (TUNEL) assay. Finally, the influence on the morphology of the heart, liver, spleen, lung and kidney of mice was test by hematoxylin and eosin (H&E) staining. Those data further support that a liver-targeting peptide CSPI-plus modified Endostar may be a potential approach for the targeting treatment of hepatocellular carcinoma.

## Results

### Antitumor activity of rES-CSP in subcutaneous xenograft models

**Figure 1:**
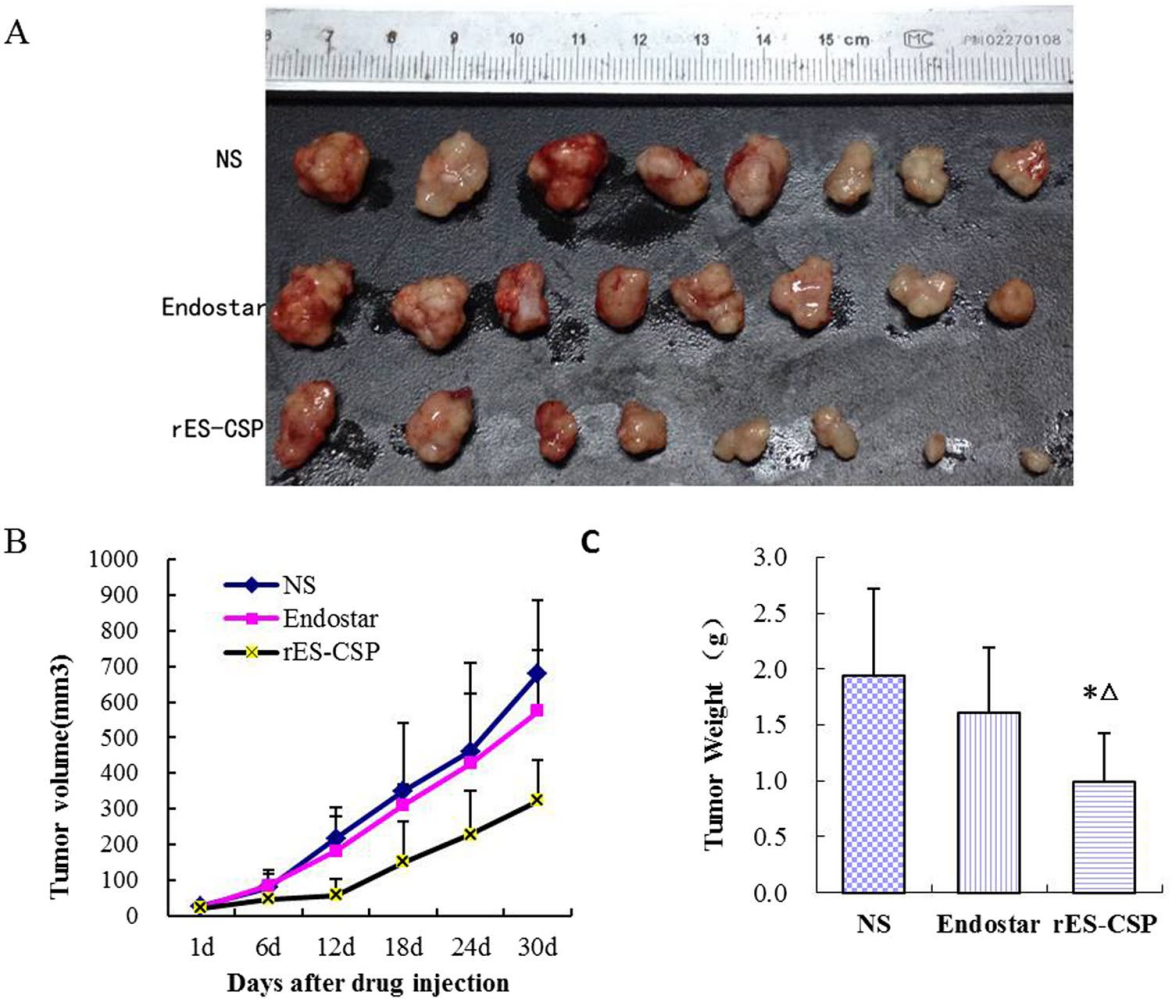
Inhibition of rES-CSP on HepG2 subcutaneous xenograft in nude mice. (**A**) Tumor size of xenograft tumor; (**B**) the growth curve of xenograft tumor; (**C**) statistics of tumor weight in different groups. (n = 8, mean ± SD, *P < 0.05. *vs* NS, ^Δ^ *P* < 0.05. *vs* Endostar).

Subcutaneous xenograft tumors of HepG2 human hepatocellular carcinoma were treated in the vicinity for four weeks, the tumor growth curve and tumor weight as shown in Fig. 1, tumors treated with rES-CSP grew very slowly, the tumor volume and tumor weight of the rES-CSP group was significantly different from that of the control group. Tumors of the Endostar group also grew slower than the control group, but Endostar inhibitory effect was obviously weaker than rES-CSP, their inhibition rate of tumor size were 16.94% and 49.05% compared with the control, respectively.

### Antitumor activity of rES-CSP in orthotopic xenograft models

**Figure 2:**
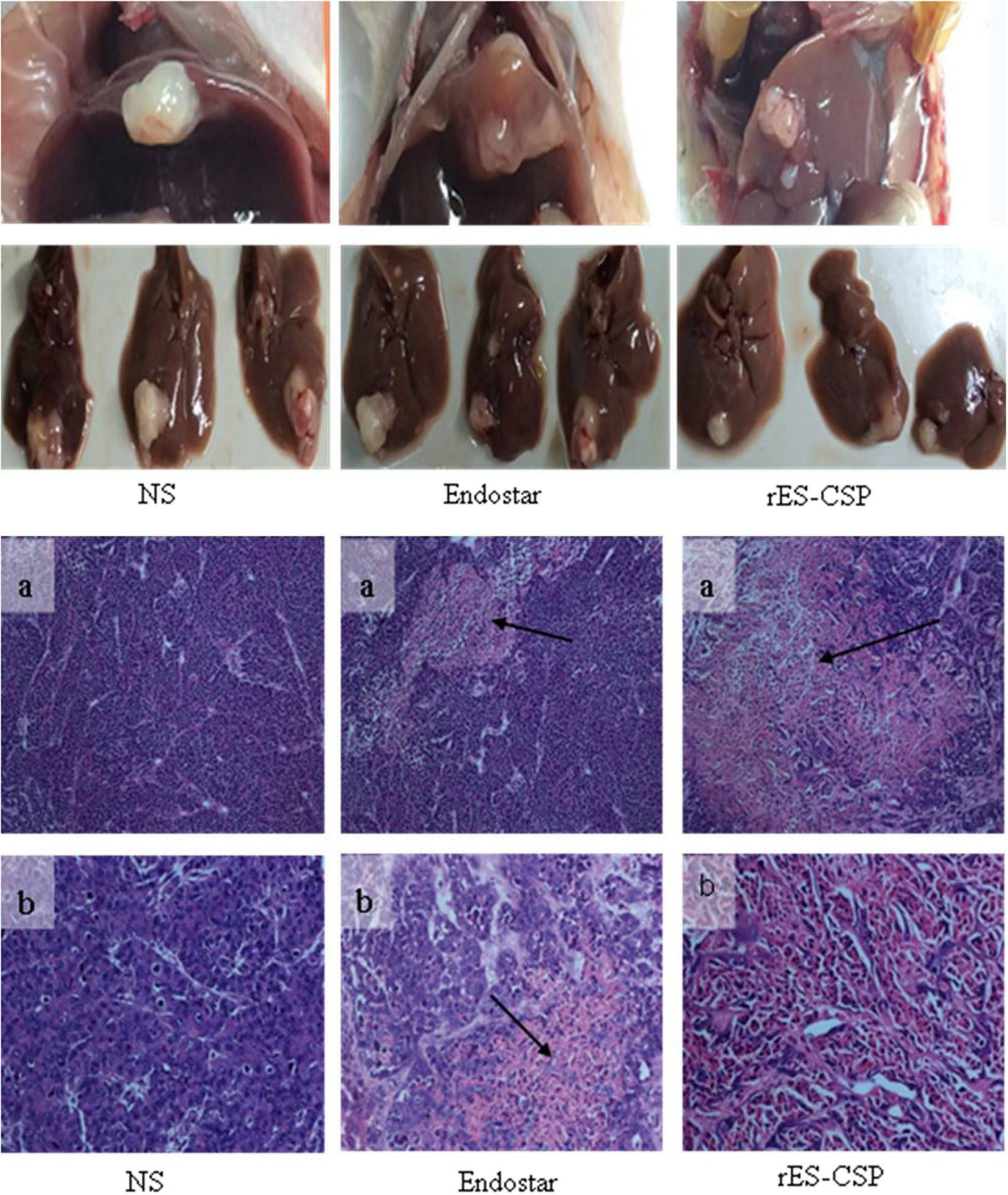
rES-CSP inhibits tumor growth in nude mice bearing orthotopic xenograft tumor. Tumor size of orthotopic xenograft tumor. Pathological changes by HE staining (arrows show cancer necrosis; a: HE × 100, b: HE × 400),

rES-CSP inhibited hepatocellular carcinoma growth in subcutaneous xenograft models of nude mice. We further analyzed the role of rES-CSP on hepatocellular carcinoma growth in orthotopic xenograft models. The results showed that rES-CSP also significantly inhibited the growth of hepatocellular carcinoma compared with Endostar and the control (Fig. 2a). The tumor inhibition rates of rES-CSP and Endostar were 39.6% and 3.8%, respectively (Table 1).

**Table1:** The effect of rES-CSP to the tumor value and tumor weight.

	Tumor Volume(mm^3^)	Tumor Weight(g)	Inhibition Ratio (%)
NS	216.34 ± 102.17	0.19 ± 0.06	—
Endostar	208.19 ± 113.17	0.18 ± 0.10	3.8
rES-CSP	130.73 ± 43.82*^,Δ^	0.09 ± 0.03*^,Δ^	39.6

(n = 8, mean ± SD, *P < 0.05. *vs* NS, ^Δ^ *P* < 0.05. *vs* Endostar).

HE staining showed that the tumor cells grew vigorously, arrangement irregularly, and they were large with more chromatin and blood vessels in the control group. Extensive necrosis and sparsely arranged nascent tumor cells were mainly observed in rES-CSP treated group, whereas there was moderate necrosis in the Endostar-treated group and mild necrosis in the control group (Fig. 2b). These results indicate that rES-CSP has the inhibitory effect on the growth of hepatocellular carcinoma.

### rES-CSP inhibited tumor angiogenesis and induced tumor cell apoptosis

**Figure 3:**
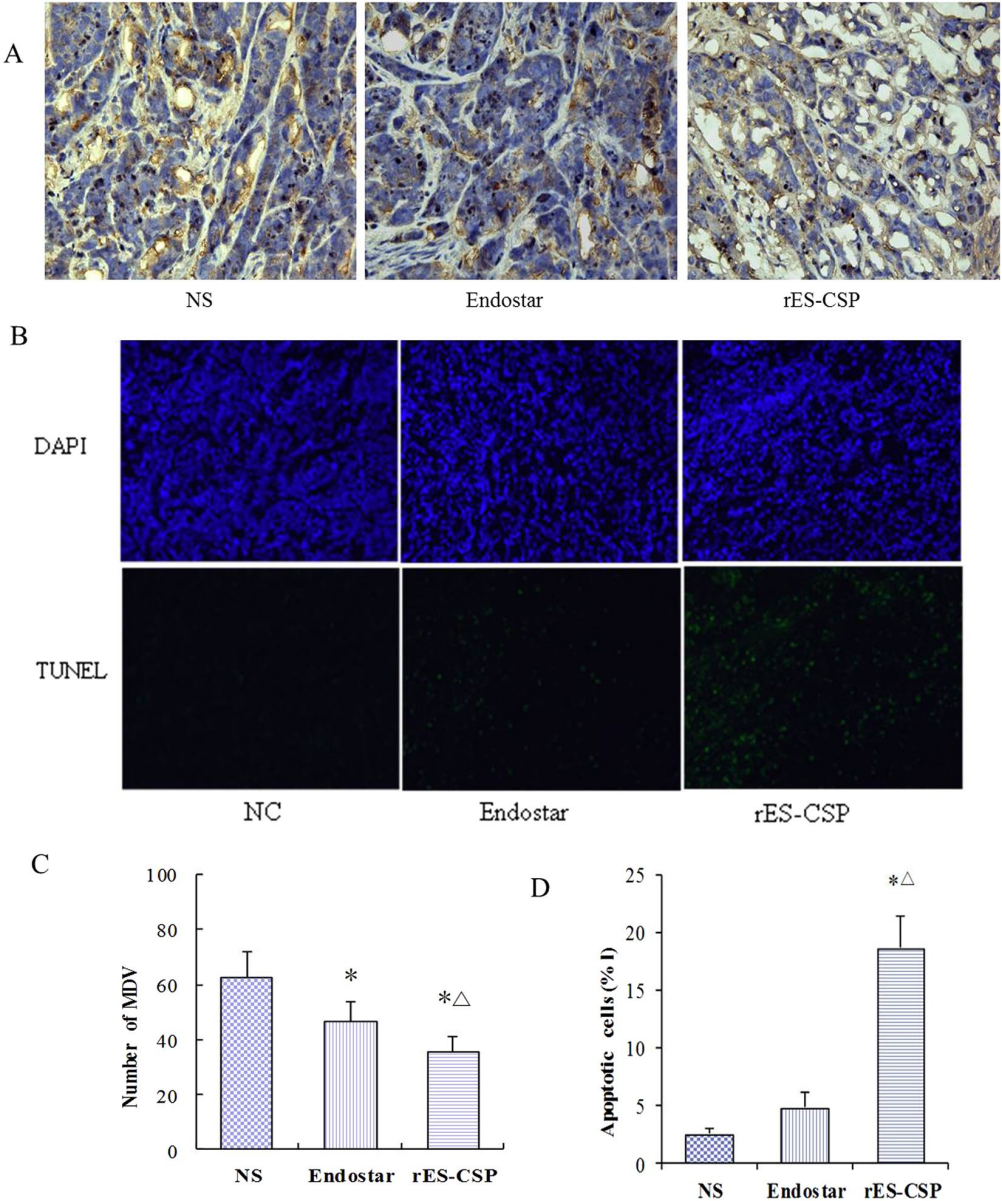
The effects of rES-CSP on tumor MVD and cell apoptosis. (**A**) MVD measurement by Immunochemical staining. Yellow color of the tumor tissues is positive CD31 (×400); (**B**) Cell apoptosis by TUNEL staining (×200). Green color is apoptotic cell stained by TUNEL; blue color is the cell nucleus stained by DAPI. (**C**) Comparision of MVD count after treatment among three groups. (**D**) Quantification of cell apoptosis from conditions in (**B**) (n = 8, mean ± SD, *P < 0.05.vs NS; ^Δ^P < 0.05 vs Endostar).

To further confirm the role of rES-CSP on angiogenesis in hepatocellular carcinoma, we stained the tumor tissues with anti-CD31 antibody. The immunohistochemistry staining indicated that the number and diameter of vascular networks treated with rES-CSP were fewer and smaller compared with those treated with Endostar or saline. MVD of hepatocellular carcinoma was determined by the vessel counts and calculated as illustrated in Fig. 3A. In tumors treated with saline and Endostar, MVD were (63.0 ± 9.32) and (47.1 ± 6.92) respectively, while MVD of those treated with rES-CSP was only (36.0 ± 5.37).

To evaluate the extent of apoptosis in tumor tissues, apoptotic cells were stained using the TUNEL method. A notable increase of apoptotic-positive cells was observed in the rES-CSP treated tumor compared with Endostar group and the saline control (Fig. 3B). Data of apoptotic indexes of the three groups were shown as follows: control group (2.48 ± 1.34%), Endostar group (4.76 ± 2.40%) and rES-CSP group (18.63 ± 4.77%) (Fig. 3D). These data suggested that CSP I-plus modified Endostar (rES-CSP) increased tumor cell apoptosis, supporting the notion that CSPI-plus modified Endostar had a synergistic effect in hepatocellular carcinoma.

### rES-CSP accumulates to liver and HCC tissue in nude mice bearing orthotopic xenograft tumor

To determine targeting efficacy of CSPI-plus modified Endostar (rES-CSP) *in vivo*, the concentration of human endostatin were measured by ELISA. Results showed that Endostar was nonspecific distribution all over the body, and the concentration of Endostar decreased rapidly. The concentration of rES-CSP was high in the kidney and blood within 5 min post injection and gradually decreased as the time elapsed, too. However, there was a significant increase in the liver and tumor tissue compared with Endostar (7.5 times and 6.7 times) at 60 min after injection, indicating that rES-CSP accumulated to liver and HCC tissue.

**Figure 4:**
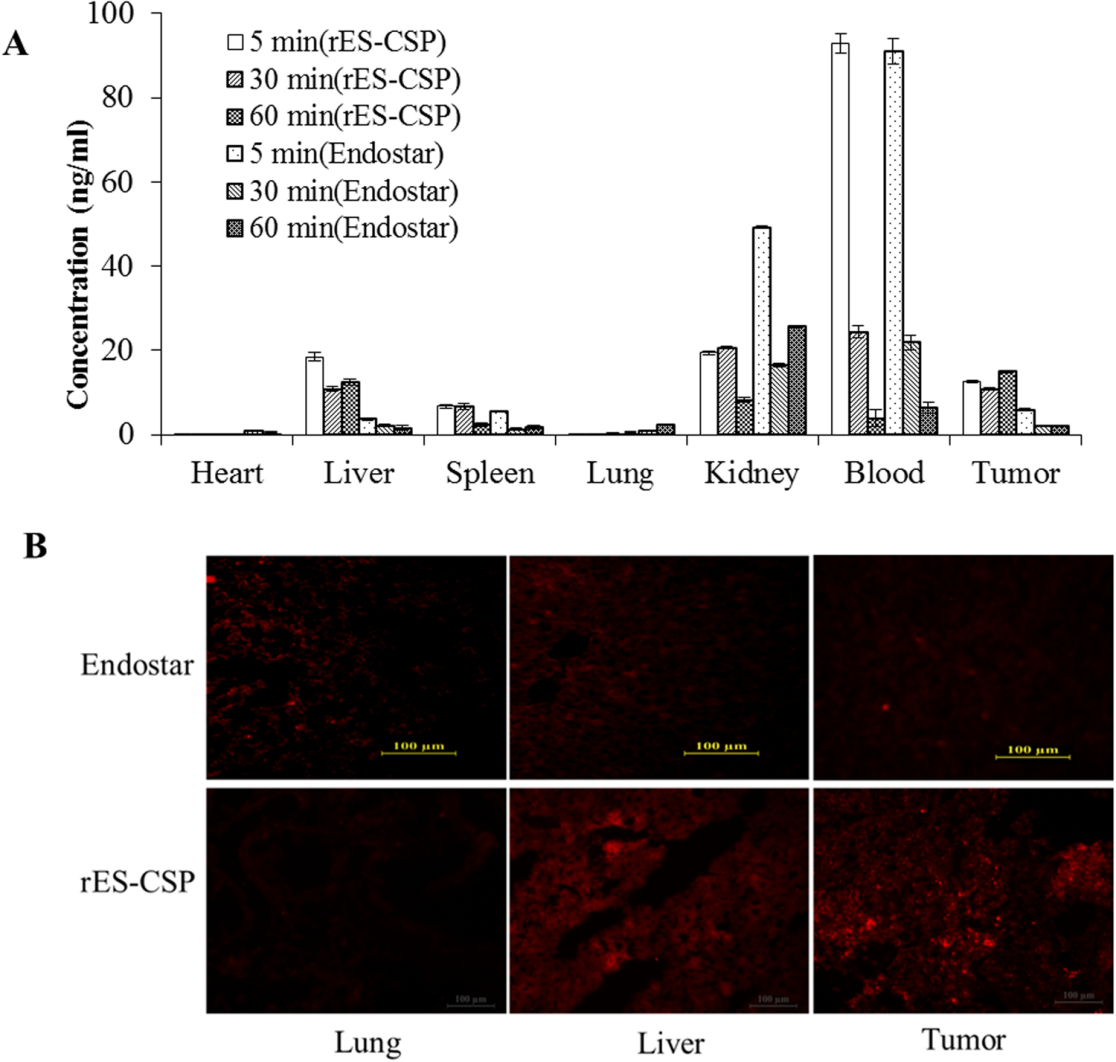
Tissue distribution comparison in mice bearing orthotopic xenograft tumor. (**A**) The concentration of human endostatin were measured at 5, 30 and 60 min time points after intravenous administration of rES-CSP and Endostar by ELISA. (**B**) Biodistribution of rES-CSP and Endostar were taken in lung, liver and hepatoma as indicated by representative endostatin immunostaining images. Red color is staining for anti-endostatin with PE.

Immunofluorescence detection of rES-CSP assessed its tissue distribution and revealed its binding specificity in mice bearing orthotopic xenograft tumor. As shown in Fig. 4, high levels of rES-CSP were detected in the normal liver tissue and tumor tissue, but not in lung tissue. At the same time, Endostar was no significant difference in the liver, tumor and lung tissues. Consistent with the above results of ELISA, CSPI-plus modified Endostar(rES-CSP) could accumulated to the liver tissue and HCC tissue.

### rES-CSP has no potential side effects in the tumor bearing mice

**Figure 5:**
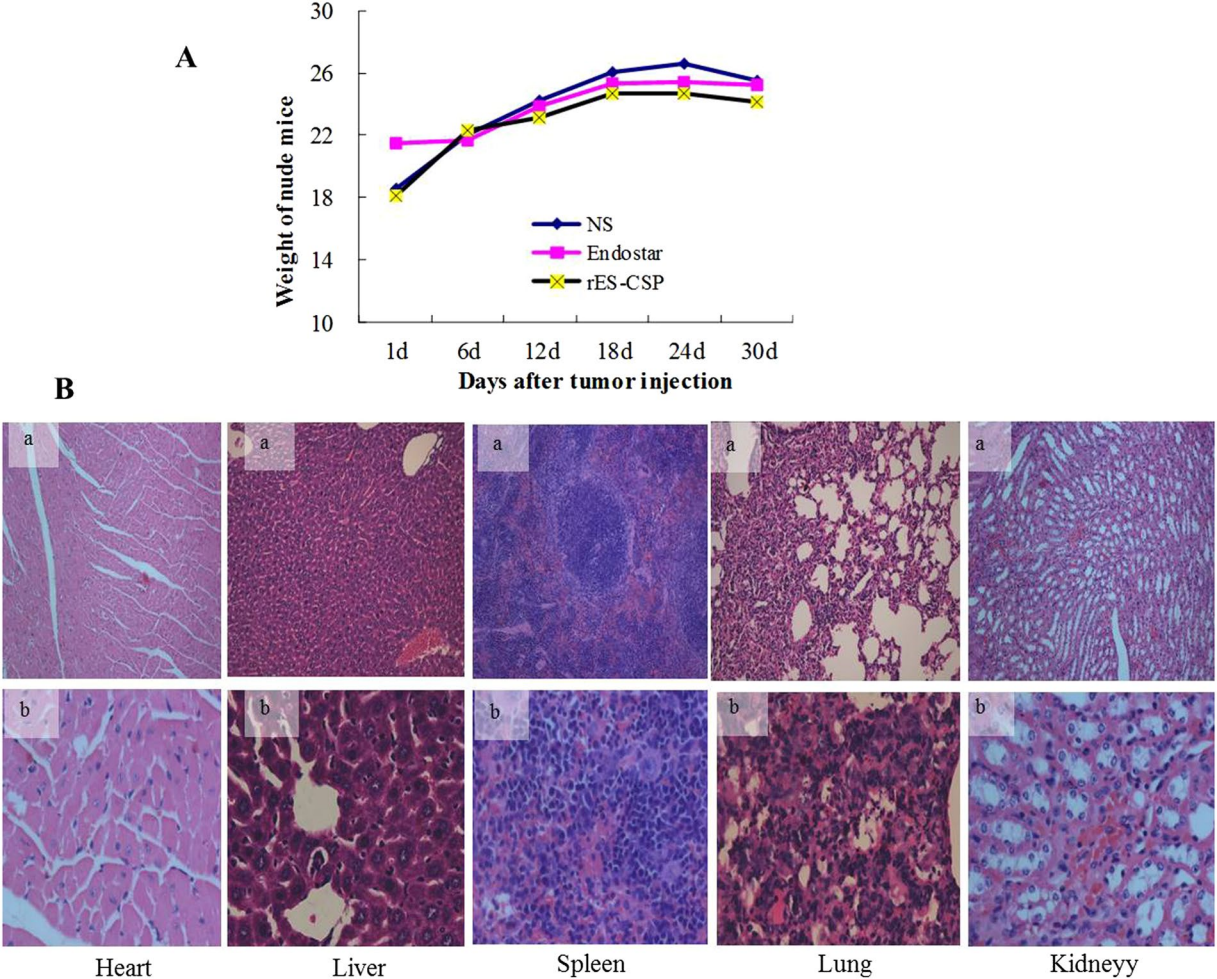
Side effects of rES-CSP was evaluated in the tumor bearing mice. (**A**) The growth state of the nude mice. (**B**) HE staining pictures of visceral tissue after rES-CSP treatment (a: HE × 100, b: HE × 400).

The toxicity of the therapeutic argent is evaluated according to the change of the body weight of the animal, which is the non-toxic reaction when the ratio of the end weight/the initial body weight is more than 0.8. The results showed that the ratio was no significant difference between the Endostar group and rES-CSP group (P > 0.05), and they were greater than 0.8(Fig. 5A).

Visceral toxicity is the most common side effect of drugs. Next, we sought to determine whether rES-CSP exerted cardiotoxicity, epatotoxicity, spleen cell toxicity as well as nephrotoxicity in tumor-bearing mice by H&E staining. Myocardial filaments were dense and neatly arranged. The hepatocytes underwent no damage as the cytoplasm displayed no vacuolation. In addition, spleen nodules was clearly visible, alveolar and glomerular morphology was normal. The kidney tissues displayed no tubular dilation and epithelial cell damaged. In a word, rES-CSP treatment had no obvious side effects.

## Discussion

Research and Development of targeted drugs is leading in the field of drug development^27^. Hepatocellular carcinoma (HCC) has a fairly high morbidity and is notoriously difficult to treat due to long latent period before detection, multidrug resistance and severe drug-related adverse effects from chemotherapy. Liver-targeting drug modified through the liver-specific ligands that can selectively deliver into HCC sites, which could be utilized to resolve the limitations of conventional chemotherapy. For example, N-lactosyl-dioleoyl phosphatidyl ethanolamine (Lac-DOPE) is a liver-specific targeting ligand via asialoglycoprotein receptors (ASGP-R), that a glycoprotein presents largely only on the liver cell surface. Lactosylated liposomes encapsulating doxorubicin (Lac-L-DOX) has significantly stronger tumor inhibitory activity compared with L-DOX and free doxorubicin, along with a higher accumulation of drug within the hepatocellular carcinoma site and greater cellular uptake by hepatocellular carcinoma cells^28^. Albert Lo used phage display to identify a novel peptide (SP94), which binds specifically to hepatocellular carcinoma cells *in vitro*. *In vivo* homing experiments show that PC94 has homing ability to tumor tissues, with a binding activity >8-fold higher than that of the control phage. With the conjugation of SP94 and liposomal doxorubicin, the targeted drug delivery system enhance the therapeutic efficacy against hepatocellular carcinoma xenografts through enhancing tumor apoptosis and decreasing tumor angiogenesis^29^.

The circumsporozoite protein (CSP) is the predominant surface antigen of Plasmodium sporozoites, a microorganism that displays highly specific and highly efficient targeting to liver, and is required for sporozoite development and invasion of hepatocytes. CSP region I-plus containing the conserved region I amino acids (KLKQP), plus the basic amino acid domain upstream from region I is proposed to be responsible for Plasmodium targeting *in vivo* by binding to the highly-sulfated heparan sulfate proteoglycans (HSPG’s) found in liver^30,31^. Liposomes, containing the amino acid sequence of CSP region I-plus that binds to hepatic heparan sulfate glycosaminoglycan, show effective targeting to liver^23,24^. Furthermore, these liposomes can deliver doxorubicin selectively to liver and HCC *in vivo* ^32^.

Our previous study suggest that CSP I-plus modified recombinant human Endostatin (rEndostatin, Endostar) could specifically target to hepatocellular carcinoma cells, to achieve higher local concentrations and showed higher anti-angiogenic capability on HUVECs tube formation assay and chick embryo CAM assay, when compared with unmodified rEndostatin given at similar doses^25^. Furthermore, CSP I-plus modified rEndostatin also specifically targeted to hepatocellular carcinoma cells and made a direct inhibition on tumor cells *in vitro* ^26^. In the present study, CSP I-plus modified recombinant human Endostatin (rES-CSP) gradually decreased as Endostar in the kidney, spleen tissue, blood, but increased significantly in the liver and hepatocellular carcinoma tissues, which demonstrated that CSP I-plus modified Endostar(rES-CSP) improved the accumulation in liver and HCC cells, and significantly enhanced the inhibitory effects on tumor growth in nude mice with subcutaneous and orthotopic xenograft models of hepatocellular carcinoma HepG2. The latter effect was supported by the massive tumor cell necrosis, significantly declined tumor MVD, and increased tumor cell apoptosis. This may be due to the specific binding of CSP I-plus to the HSPGs receptor in hepatocytes and HCC cells. rES-CSP was further transported into the HCC cells through a receptor mediated active targeting, demonstrating the advantage for the treatment of HCC.

The mostly causes of death of cancer patients are liver or kidney failure. Meanwhile current clinical use of anti-tumor chemotherapy drugs have intrinsically toxic of different levels to the heart and lung and kidney, which significantly accelerated cancer patients functional organ failure and limit the dose of chemotherapy drugs and even the use of. Besides toxicity, chemotherapy can lead to side-effects like fatigue, hair loss and weight loss. We evaluated the toxicity of rES-CSP according to the changes of the body weight and the visceral organ histomorphology, and confirmed that rES-CSP has no significant side effects, can be as a safe candidate drug for anti-HCC. The results in this study were consistent with previous reports^16,33^.

In summary, rES-CSP increased antitumor activity, improved selectivity and had no significant side-effects in BALB/c nude mice with hepatocellular carcinoma. Experiments are ongoing in our laboratory to determine whether CSP I-plus could increase other antitumor domains (e.g. interferon α, interleukin-24, tumor necrosis factor α, etc) homing to hepatocellular carcinoma cells and their biologic activity. In addition, the detailed mechanisms for homing to liver and the directly efficacy of CSP I-plus-rEndostatin in treatment of hepatocellular carcinoma may need further studies. rES-CSP may be a potential therapeutic agent for human HCC, and CSP I-plus thus has significant clinical potential to improve the targeting treatment of of the liver diseases.

## Materials and Methods

### Materials

Recombinant human endostatin (Endostar) was purchased from Shandong Simcere-Medgenn Bio-Pharmaceutical Co.,Ltd and dissolved in phosphate buffered saline(PBS) at a concentration of 3.0 g/L. The fusion proteins (rES-CSP) were prepared after expressing and purification by our laboratory and diluted in PBS at a concentration of 3.0 g/L. Reagents were sterilized by passing through a 0.22 µm filter and stored at −20 °C. Human Endostatin ELISA kit was purchased from RayBiotech. Antibodies to CD31 and Endostatin were purchased from Abcam. The *in situ* cell death detection kit was purchased from Roche.

### Animals and cell culture

Female athymic BALB/c nu/nu mice(body weight 18–22 g, 4–6 weeks old) were obtained from the Guangdong Medical Laboratory Animal Center(GDMLAC). Mice were raised under specific pathogen-free conditions. Principles of laboratory animal care were followed and all procedures were conducted according to the guidelines established by the Institutional Animal Care and Use Committee at the Chinese Academy of Medical Science and National Institutes of Health. Every effort was made to minimize suffering. This study was approved by the Animal Experiment Committee of Guangdong Pharmaceutical University. Human hepatocellular carcinoma cell HepG2 was purchased from Shanghai Cell Biology Institutes of China and cultured in DMEM (supplemented with 10% fetal bovine serum, 100 µg/ml streptomycin and 100 U/ml penicillin) in humidified air containing 5% CO_2_ at 37 °C

### Tumor growth assays in subcutaneous xenograft nude mice models

Briefly, approximately 1 × 10^7^ HepG2 cells were collected and suspended in 0.1 ml PBS, and injected subcutaneously into the beneath flank of BALB/c nude mice. The subcutaneous tumors were allowed to grow up and tumor volumes were measured with caliper. When the maximum diameter of tumor was reached approximately 5 mm, mice were treated with 15 mg/kg Endostar or rES-CSP in the vicinity of the tumor once a day for four weeks. Equal volume of saline was injected in the control mice. The longer (L) and the width (W) of the tumor were measured every 6 days in period. The tumor volume was calculated via the formula V = (LW^2^)/2. Mice were sacrificed on the third day after the last administration. Tumors were excised, weighed and photoed.

### Tumor growth assays in orthotopic xenograft nude mice models

When the maximum diameter of subcutaneous xenograft tumors were reached approximately 1 cm, tumors were removed, minced into small pieces about 2 mm^3^ and surgically implanted in the livers of 24 nude mice. The animals were randomly divided into three groups (n = 8). On day 7 to 37 after surgery, 15 mg/kg Endostar and rES-CSP were injected into mice once a day by tail vein respectively, and the mice were injected saline as negative control. All mice were sacrificed, and tumors were excised, weighed and photoed on day 38. And the weights of the tumors were measured to evaluate the anti-tumor effects. The inhibition rates of tumors with the formula:

$${\rm{inhibition}}\,{\rm{rates}}\,( \% )=[[{{V}}_{{\rm{tumor}}({\rm{control}})}-{{V}}_{{\rm{tumor}}({\rm{treated}})}]/{{V}}_{{\rm{tumor}}({\rm{control}})}{\rm{.}}$$

### Histopathology

The heart, liver, spleen, lung, kidney and tumor tissue of mice bearing orthotopic xenograft tumor were respectively harvested at the end of the experiment on day 38 post-surgery. Samples were excised, fixed with 10% neutral phosphate-buffered formalin and embedded in paraffin. Continuous sections (5 µm) were obtained and stained with hematoxylin and eosin (H&E) for histomorphometric analyses.

### Tumor microvessel density (MVD) measurement

To investigate the effects of the fusion protein on tumor angiogenesis, tumor microvessel density (MVD) was quantified using anti-CD31 immunostaining. Tumor sections were incubated with monoclonal rabbit anti-CD31 antibody (Abcam) at a dilution of 1:50 overnight at 4 °C and then rinsed three times, followed by HRP- conjugated anti-Rabbit lgG(H + L) for 1 h. Immuno-reactivity was detected by the standard avidin–biotin immunoperoxidase method. Counterstaining with Meyer’s hematoxylin was then performed for 5 min. Images were photographed under light microscope (Olympus, Japan). Tumor microvessel density was measured using Image-Pro Plus 6.0 software.

### *In situ* cell death detected by TUNEL assay

Apoptosis-induced DNA fragmentation was determined by the transferase mediated deoxyuridine triphosphate (dUTP)-digoxigenin nick end-labeling (TUNEL) assay in accordance with the manufacturer’s instructions. Briefly, tumor sections were dewaxed and rehydrated according to standard protocols, and permeabilized with 0.1% Triton X–100 in 0.1% sodium citrate for 8 min. TUNEL staining was done using the *in situ* cell death detection kit (Roche) and the nuclei were stained with DAPI for 10 min. The numbers of TUNEL-positive cells and the total cells were captured with fluorescence microscope (Olympus). Cell apoptosis was measured using Image-Pro Plus Software.

### Detection of biodistribution by ELISA

The biodistribution of rES-CSP *in vivo* was determined using human Endostatin ELISA kit (RayBiotech). Orthotopic xenograft models were established for four weeks, serum and the supernatant of five internal organs were collected at 15, 30, 60 min after single-dose tail vein injection, and measured by ELISA following the manufacturer’s instructions. In brief, 100 µl of diluted serum or supernatant samples were loaded in pre-coated 96-well strip microplate and incubated at RT for 2 h. Diluted HRP-Streptavidin solution was added to each designated well at 37 °C for 1 h. TMB substrate buffer was added to each well, and stopped after 10 min by treating with 100 µl of stop solution. The ELISA results were expressed as the OD values measured at 450 nm by a microplate reader.

### Detection of binding specificity by immunofluorescence

Internal organs of orthotopic xenograft mice were collected at 60 min after single-dose tail vein injection, embedded in O.C.T.and cut into 5 µm frozen sections. The sections were treated with 10 mM citrate buffer (pH 6.0) at 95 °C to retrieve antigens and blocked with 10% BSA (Albumin from bovine serum), stained with anti-ES polyclonal antibody, and then PE-conjugated anti-mouse lgG antibody. Red fluorescent protein-positive foci were analyzed by fluorescence microscopy. The integrated absorbance was quantitated by Image-Pro Plus software.

### Statistical analysis

Statistical analysis of data was processed with SPSS13.0 software. All quantitative data were presented as mean ± SD and analyzed by one-way ANOVE for multiple comparisons. Results with *p* < 0.05 were considered statistically significant.

## References

[1] Iliescu L (2014). Management of hepatocellular carcinoma - experience of a single center. Chirurgia (Bucur)..

[2] Fitzmorris P, Shoreibah M, Anand BS, Singal AK (2015). Management of hepatocellular Carcinoma. J Cancer Res Clin Oncol..

[3] Varo PE, Castroagudin JF (2010). The future of liver transplantation. Trannsplant Proc..

[4] Andreou A (2013). Improved long-term survival after major resection for hepatocellular carcinoma: a multicenter analysis based on a new definition of major hepatectomy. J Gastrointest Surg..

[5] Cheng Z (2015). Risk factors and management for early and late intrahepatic recurrence of solitary hepatocellular carcinoma after curative resection. HPB (Oxford)..

[6] Goyal A, Wang S, Siegel AB (2012). Prognostic value of vascular endothelial growth factor levels in patients with hepatocellular carcinoma. J Clin Oncol..

[7] Cheng AL (2009). Efficacy and safety of sorafenib in patients in the Asia-Pacific region with advanced hepatocellular carcinoma: a phase III randomised, double-blind, placebo-controlled trial. Lancet Oncol..

[8] Yokoyama Y, Ramakrishnan S (2004). Addition of integrin binding sequence to a mutant human endostatin improves inhibition of tumor growth. Int J Cancer..

[9] Sund M (2005). Function of endogenous inhibitors of angiogenesis as endothelium-specific tumor suppressors. Proc Natl Acad Sci. USA.

[10] Hajitou A (2002). The antitumoral effect of endostatin and angiostatin is associated with a down-regulation of vascular endothelial growth factor expression in tumor cells. FASEB J..

[11] Cui R (2007). Signal transduction mediated by Endostatin directly modulates cellular function of lung cancer cells *in vitro*. Cancer Sci..

[12] Yokoyama Y, Sedgewick G, Ramakrishnan S (2007). Binding of endostatin to human ovarian cancer cells inhibits cell attachment. Int J Cancer..

[13] Guo H (2016). Endostatin inhibits the growth and migration of 4T1 mouse breast cancer cells by skewing macrophage polarity toward the M1 phenotype. Cancer Immunol Immunother.

[14] Foguer K, Braga Mde S, Peron JPS, Bortoluci KR, Bellini MH (2016). Endostatin gene therapy inhibits intratumoral macrophage M2 polarization. Biomed Pharmacother..

[15] Jiang LP (2009). N-terminal modification increases the stability of the recombinant human endostatin *in vitro*. Biotechnol Appl Biochem..

[16] Huang W (2016). The efficacy and safety of endostar combined with taxane- based regimens for HER-2-negative metastatic breast cancer patients. Oncotarget..

[17] Chen X (2016). Endostatin combined with radiotherapy suppresses vasculogenic mimicry formation through inhibition of epithelial-mesenchymal transition in esophageal cancer. Tumour Biol..

[18] Chen Y (2011). Cholesterol sequestration by nystatin enhances the uptake and activity of endostatin in endothelium via regulating distinct endocytic pathways. Blood..

[19] Shockley TR (1991). Penetration of tumor tissue by antibodies and other immunoproteins. Ann N Y Acad Sci..

[20] Lo A, Lin CT (2008). Wu HANC. Hepatocellular carcinoma cell-specific peptide ligand for targeted drug delivery. Mol Cancer Ther..

[21] Coppi A (2011). The malaria circumsporozoite protein has two functional domains, each with distinct roles as sporozoites journey from mosquito to mammalian host. J Exp Med..

[22] Plassmeyer ML (2009). Structure of the Plasmodium falciparum circumsporozoite protein, a leading malaria vaccine candidate. J Biol Chem..

[23] Longmuir KJ, Robertson RT, Haynes SM, Baratta JL, Waring AJ (2006). Effective targeting of liposomes to liver and hepatocytes *in vivo* by incorporation of a Plasmodium amino acid sequence. Pharm Res..

[24] Robertson RT, Baratta JL, Haynes SM, Longmuir KJ (2008). Liposomes incorporating a Plasmodium amino acid sequence target heparan sulfate binding sites in liver. J Pharm Sci.

[25] Ma Y, Jin X-B, Chu F-J, Bao D-M, Zhu J-Y (2014). Expression of liver-targeting peptide modified recombinant human endostatin and preliminary study of its biological activities. Appl Microbiol Biotechnol..

[26] Bao D, Jin X, Ma Y, Zhu J (2015). Comparison of the Structure and Biological Activities of Wild-type and Mutant Liver-targeting Peptide Modified Recombinant Human Endostatin (rES-CSP) in Human Hepatocellular Carcinoma HepG2 Cells. Protein Pept Lett..

[27] Zhang X (2016). Drug delivery system targeting advanced hepatocellular carcinoma: Current and future. Nanomedicine..

[28] Zhou X (2012). Lactosylated liposomes for targeted delivery of doxorubicin to hepatocellular carcinoma. Int J Nanomedicine..

[29] Lo A, Lin C-T, Wu H-C (2008). Hepatocellular carcinoma cell-specific peptide ligand for targeted drug delivery. Mol Cancer Ther..

[30] Armistead JS, Wilson IB, van Kuppevelt TH, Dinglasan RR (2011). A role for heparan sulfate proteoglycans in Plasmodium falciparum sporozoite invasion of anopheline mosquito salivary glands. Biochem J..

[31] Tsai MS, Baratta JL, Longmuir KJ, Robertson RT (2011). Binding patterns of peptide- containing liposomes in liver and spleen of developing mice: comparison with heparan sulfate immunoreactivity. J Drug Target..

[32] Longmuir KJ, Haynes SM, Baratta JL, Kasabwalla N, Robertson RT (2009). Liposomal delivery of doxorubicin to hepatocytes *in vivo* by targeting heparan sulfate. Int J Pharm..

[33] Biaoxue R, Xiguang C, Hua L, Wenlong G, Shuanying Y (2016). Thoracic perfusion of recombinant human endostatin (Endostar) combined with chemotherapeutic agents versus chemotherapeutic agents alone for treating malignant pleural effusions: a systematic evaluation and meta-analysis. BMC Cancer..

